# Successful myomectomy in early pregnancy for a large asymptomatic uterine myoma: case report

**DOI:** 10.11604/pamj.2016.24.228.9890

**Published:** 2016-07-13

**Authors:** Pawan Jhalta, Sonam Gialchhen Negi, Vikas Sharma

**Affiliations:** 1 Department of OBG Regional Hospital Reckong Peo Kinnaur, HP, India 172107; 2 Department of Radiology Regional Hospital Reckong Peo Kinnaur, HP, India

**Keywords:** Pregnancy, uterine myoma, myomectomy

## Abstract

The decision of myomectomy is not usually taken by OBG specialist for uterine fibroids during pregnancy because of its complications which may become hazardous at times. This is why it is generally delayed until after delivery. The current case was a large, asymptomatic subserous uterine myoma diagnosed during pregnancy by ultrasound and successfully managed by antepartum myomectomy retaining the fetus alive in utero at 13 -14 weeks gestation. At term, the patient had spontaneous vaginal delivery of 3 kg male child. This case demonstrates that myomectomy during pregnancy in special circumstances in selected cases to prevent forthcoming events adversely affecting mother and fetus can be considered.

## Introduction

The prevalence of uterine myomas during pregnancy is estimated to be 0.3% to 2.6%, of which 10% result in pregnancy complications [1]. Although leiomyomas are usually asymptomatic during pregnancy however they may complicate its course. The complications include first trimester losses, pressure symptoms caused by the myoma on the mother and fetus, pain of “red degeneration”, premature labor, premature rupture of membranes, malpresentation, retained placenta, postpartum hemorrhage and uterine torsion [2]. The size, location, number of fibroids and their relation to the placenta are critical factors. Ultrasound scanning plays a central role in diagnosing and monitoring fibroids during pregnancy and in determining the relative position of the fibroids to the placenta. The management of leiomyoma during pregnancy is medical, but, in rare circumstances, surgical intervention and myomectomy may be required [3].

## Patient and observation

**Figure 1:**
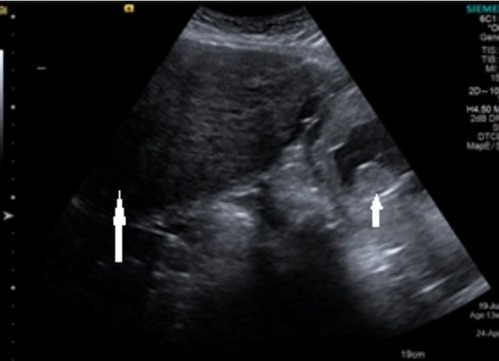
Ultrsonographic picture showing intrauterine fetus (short arrow) with large mass (long arrow) in relation to fundus

**Figure 2:**
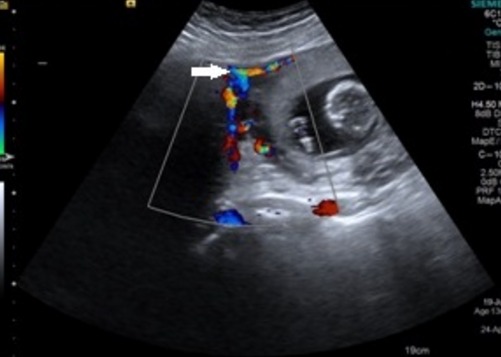
Ultrsonographic picture showing bridging vessel (white arrow) between the mass lesion and gravid uterus

**Figure 3:**
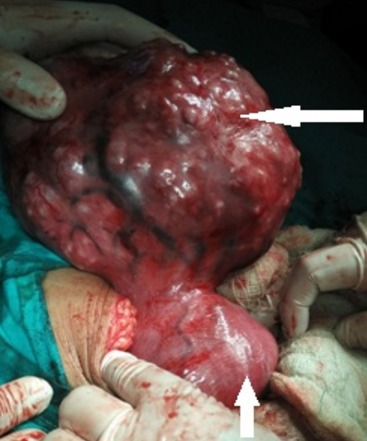
Intraoperative picture showing gravid uterus (short arrow) with large subserous pedunculatedmyoma (long arrow)

**Figure 4:**
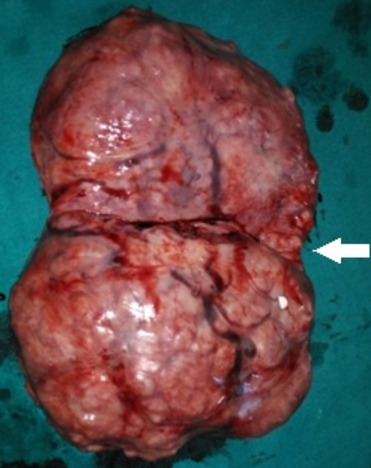
The large 4.5 kg fibroid with its pedicle site (arrow)

A 34 year old pimigravida presented in our hospital on 20 October 2015 at period of gestation 13 weeks 2days for routine antenatal examination. On examination her height of uterus was found to be 30-32 weeks gestational size. On vaginal examination we could feel the uterus separately of about 12-14 weeks gestational size, beside this a huge mass of about 15 cm diameter filling whole abdomen was felt separately. Ultrasonography showed an intrauterine viable fetus of 14 weeks 2 days Gestation with a large well defined hypoechoic abdominopelvic mass measuring 16x10 cm with minimum vascularity seen which was extending from right adenexa to right side of abdominal cavity up to right hypochondrium (Figure 1). A bridging vessel was seen extending from fundus of uterus to the mass lesion so 1st possibility of degenerated pedunculated fibroid was kept however right ovary was not seen separately from the mass lesion so the possibility of ovarian mass was also not ruled out (Figure 2). Patient was counselled regarding all possible outcomes of pregnancy along with this mass and she agreed to undergo exploratory laparotomy. On laparotomy a huge subserosal pedunculated fibroid arising from right fundic region of size 16x12x10 cm was found and removed (Figure 3, Figure 4). Following leiomyoma removal haemostasis was carefully achieved. Fetal monitoring by ultrasonography was carried out immediately after surgery and fetus was found viable. Postoperative period was uneventful and patient was given tocolysis for 3 days with uterine relaxants and micronized progesterone was continued for 4 weeks. Patient was discharged on 10th post operative day and followed up using ultrasonography 4 weekly till 38 weeks at which patient was admitted in hospital. Patient went into spontaneous labour at 39 weeks 1 day and delivered vaginally a male child weighing 3 kg with apgar score of 8 and 10. Mother and baby was discharged after 48 hours from the hospital.

## Discussion

Controversy persists among reports of myomectomy being performed during pregnancy. The management of uterine leiomyoma during pregnancy is largely expectant and its surgical removal is generally delayed until after delivery [4]. Mortality and morbidity are slightly higher in myomectomy in the gravid uterus as compared to non-gravid uterus [2]. There is increased vascularity of the gravid uterus, thus myomectomy performed in pregnancy has been reported to be associated with greater risk of haemorrhage and the need for blood transfusion [5]. Additional to the general risks of myomectomy is the risk of abortion which occurs in 18% to 35% of cases [6]. An incomplete abortion may also result in severe endometritis especially if a communication channel to the recently enucleated fibroid bed is present [2].

In our case 34 year old lady presented to us in a remote geographical location for her antenatal examination at 13 weeks 2 days and was diagnosed with large abdominopelvic mass on ultrasonography. Although our patient was asymptomatic but we could not categorically rule out ovarian origin of the mass. After explaining the risks associated with such a large mass patient gave consent for exploratory laparotomy and myomectomy was done. The subserosal location made it easier to remove the fibroid and hypercoagulability of pregnancy contributed to the ease in achieving hemostasis which further contributed to safety of the procedure.

A reported myomectomy during early pregnancy was in women from Latin America [7] presented with progressively worsening lower abdominal pain, needed laparotomy at 15 weeks gestation a pedunculated myoma showing degenerative changes in the fundus of the uterus was excised successfully and the pregnancy progressed normally. Other myomectomies from Singapore were during cesarean section [8]. Mollica et al. [9] conducted a prospective study of 106 pregnant women with uterine myomas who were admitted with recurrent abdominal pain. This study shows that regardless of gestational age, the outcomes for all women who underwent myomectomy (n = 18) was superior to those managed conservatively in terms of pregnancy loss (0% versus 13.6%), premature rupture of membranes (5.6% versus 22.7%), preterm labour (5.6% versus 21.6%) and post-cesarean hysterectomy (0% versus 4.5%). However in patients like our own a timely and well planned myomectomy can be an option offered to the patient, avoiding morbidity and mortality associated with emergency procedures.

## Conclusion

In conclusion the decision to perform a myomectomy during pregnancy should be based upon the fibroid size, location and its rapid growth to prevent various possible forthcoming adverse events. Therefore, a carefully planned myomectomy in huge myomas in selected cases is an appropriate low morbidity option which can be offered to the patient.
